# Editorial for Special Issue “Advanced Materials in Catalysis and Adsorption”

**DOI:** 10.3390/ma16072895

**Published:** 2023-04-05

**Authors:** Ilya V. Mishakov

**Affiliations:** Department of Materials Science and Functional Materials, Boreskov Institute of Catalysis, Lavrentieva Ave, 5, 630090 Novosibirsk, Russia; mishakov@catalysis.ru

This Special Issue aims to cover the latest research on the design and development of advanced materials for adsorption and catalytic applications. The synthesis of various carbon nanomaterials (CNMs), including carbon nanofibers (CNFs), carbon nanotubes (CNTs), graphene-like materials, and carriers (Sibunit), is of particular interest [1,2,3,4,5,6,7,8]. The functionalization of CNMs with heteroatoms (i.e., nitrogen [1,6,7,8] and boron [3]) is an effective method for boosting the applicability of carbon nanomaterials as catalytic substrates. Another group of the designed materials belongs to the oxide-based supports, including zeolite catalysts [9], composite catalytic materials [5,10,11,12], mixed Mn-Zr-Ce-O oxides [13], and perovskite-like materials [14]. In many cases, the active component of the developed catalysts is represented by precious metals—Pd [1,7], Pt [5,11], Rh [10,11], and Ru [11]—and other metals such as Ag [15], Au [7,9], and their alloys.

Advanced catalytic materials have been developed for diverse types of heterogeneous catalytic reactions, such as the hydrodechlorination of chloroaromatics [1]; the dehalogenation of halogenated hydrocarbons [2]; dechlorination via catalytic pyrolysis [1,2,4]; the catalytic coupling of CH_4_ [14]; the catalytic processing of hydrocarbons and their mixtures into synthesis gas [10,11] or CNT and CNF materials [2,5,6,8]; the oxidation of carbon monoxide [13]; the hydrogenation of organic compounds [7]; and selective oxidation [9]. Such advanced materials can be also used for electrocatalytic applications involving the oxygen evolution reaction (OER) [12] and the electrocatalytic reduction of carbon dioxide (CO_2_RR) [15]. Researchers’ attention has been especially drawn to environmental protection, where advanced materials and adsorbents are highly demanded for the processing of waste components [1,16,17,18] and pharmaceuticals [17], the (photo)degradation of dyes [16,18], the catalytic decomposition of chlorinated hydrocarbons [1,2,4], wastewater treatment [1,18], and the abatement of CO-containing gases [13].

The functionalization of various CNMs for further application as catalytic supports and sorbents has attracted a great deal of research attention. A new method of synthesizing a boron-doped graphene material using a template concept has been proposed in [3]. The produced B-doped graphene material (composed of three to eight monolayers) is characterized by a boron concentration of about 4 wt.% and a high specific surface area (SSA) of 800 m^2^/g. The post-functionalization of carbon nanofibers with nitrogen using melamine was described in [6]. Therein, the authors revealed that the preliminary acidic treatment of CNFs permits an increase in the amount of N-doping (from 1.4 to 4.3 wt.%), while preserving the structure and consistency of the initial carbon filaments. Diazonium salts can also be effectively used for the surface modification of carbonaceous supports (Sibunit) used as carriers for Pd- and Pd-Au catalysts for furfural hydrogenation [7]. The authors concluded that the modification of Sibunit with various functional groups leads to changes in the hydrophobic/hydrophilic and electrostatic properties of the surface, thus influencing its selectivity.

On the other hand, the “one-pot” synthetic approach to obtaining N-functionalized carbon nanofilaments was successfully implemented in [1,8]. Acetonitrile [1] and NH_3_ [8] were employed as the N-containing co-reagents in CVD-synthesis, thus facilitating the introduction of nitrogen in one stage. It was found [8] that the number of bamboo-like and spherical structures induced by the incorporation of nitrogen into the CNTs’ structure depends on the temperature (T_opt_ = 650–700 °C) and the NH_3_ concentration in the reaction mixture (the optimal composition was C_3_H_8_/NH_3_ = 50/50%).

The carbon erosion or metal dusting (MD) of bulk Ni-M and Co-M alloys can be used to produce carbon nanofibers and CNF-based composites [1,2,4,5,6]. The carbon erosion of nickel alloys during the catalytic pyrolysis of organic compounds with the formation of carbon nanofibers in a flow-through reactor and under reaction conditions in a close volume (Reactions under Autogenic Pressure at Elevated Temperature (RAPET)) has been extensively studied in [2]. The efficiency of using the ferromagnetic resonance (FMR) method to monitor the appearance of catalytically active nickel was demonstrated. The controllable synthesis of CNF and Co-Pt/CNF composites via ethylene decomposition over Co-Pt (0–100 at.% Pt) microdispersed alloys was described in [5]. The authors revealed the impact of the Pt content in Co_1−x_Pt_x_ alloys on their activity in CNFs and found that the addition of 15–25 at.% Pt to a cobalt catalyst leads to a three- to five-fold increase in productivity [5].

Furthermore, chlorinated hydrocarbons can also serve as a carbon source in catalytic pyrolysis reactions [1,4]. Thus, the research on trichloroethylene (TCE) decomposition over a microdispersed Ni-W alloy (4 wt.% W) revealed that the addition of tungsten results in an enhancement of Ni productivity (1.5–2 times) toward the generation of carbon filaments with a unique segmented structure and a high SSA of 374 m^2^/g [4]. A similar approach to synthesizing CNFs via the catalytic processing of TCE (a component of organochlorine wastes) over a Ni catalyst was realized in [1]. The segmented CNFs produced were shown to be an effective adsorbent for the decontamination of water containing traces of 1,2-dichlorobenzene (1,2-DCB). The deposition of Pd nanoparticles (1.5 wt.%) on the surface of the CNFs allowed the authors to create an adsorbent with a catalytic function. It was found that the repeated use of regenerated adsorbent catalysts for the purification of aqueous solutions ensures the almost complete removal of 1,2-DCB.

Oxide-based materials with advanced features are also within the scope of the collected papers [9,13,14]. A comprehensive crystallochemical analysis of the possible structure of planar defects in Sr_2_TiO_4_-layered perovskite (a prospective catalyst for CH_4_ oxidative coupling) was reported in [14]. The authors established a relationship between the concentration of planar defects and the non-stoichiometry of the Sr_2_TiO_4_ phase. The presence of defects is thought to lead to the enrichment of the surface with Sr, which might serve as an explanation for the catalytic activity of Sr_2_TiO_4_ in the considered reaction. In [13], researchers investigated the catalytic performance of MnO_x_-CeO_2_, MnO_x_-ZrO_2_, and MnO_x_-ZrO_2_-CeO_2_ with a molar ratio of Mn/(Zr + Ce + Mn) = 0.3 in the CO oxidation reaction. The authors found that the thermal stability of the catalysts is determined by the decomposition temperature of the solid solution of Mn_x_(Ce,Zr)_1−x_O_2_. The high CO oxidation activity of the samples was shown to be correlated with the presence of oxygen vacancies and the content of easily reduced fine MnO_x_ particles. The “metal-support” interaction and its impact on the performance of gold-supported zeolitic catalysts were investigated in [9]. Three-dimensional HBeta and layered two-dimensional MCM-36 were used as supports for gold and then studied with respect to the oxidation of glucose to gluconic acid (with O_2_ and H_2_O_2_). The important roles of the porosity of the zeolite supports, the accumulation of a negative charge on the Au nanoparticles, and the impact of the oxidant (O_2_ or H_2_O_2_) on the nature of the reaction’s limiting step were revealed.

This Special Issue also includes studies related to the design of novel materials for electrocatalytic reactions. In [12], the researchers fabricated a high-performance and low-cost porous TiN electrocatalyst for the oxygen evolution reaction (OER). The authors applied a method of the thermal nitriding of Ti to prepare a novel TiN-Ti support, which was found to yield better dispersion of the active component (IrO_x_), lower ohmic resistance, and more accessible catalytic active sites on the catalytic interface. At the same time, the study in [15] focused on searching for a selective electrocatalyst for the CO_2_ reduction reaction (CO_2_RR). Accordingly, the researchers fabricated a series of Ag-Zn alloy catalysts using magnetron co-sputtering and explored their electrocatalytic performance in terms of the CO_2_RR process. The synergistic effects of Ag and Zn on Ag_5_Zn_8_ and AgZn_3_ catalysts were established. The authors concluded that the activity and selectivity of CO_2_RR are highly dependent on the element ratio and phase composition in the Ag-Zn alloyed system.

The composite structured catalysts M/Ce_0.75_Zr_0.25_O_2_/Al_2_O_3_/FeCrAl (M = Pt, Rh, Ru) were designed in [10,11] for the catalytic processing of light hydrocarbons into synthesis gas. The catalysts are composed of a high-heat-conducting FeCrAl block covered by θ-Al_2_O_3_ and a layer of ceria–zirconia mixed oxide with deposited 2–3 nm sized Pt, Rh, or Ru nanoparticles. Reformates were produced via the reformation of propane steam over the designed catalysts at 600 °C; the reformates contained ~65 vol.% of H_2_, which can be used as a fuel for solid oxide fuel cells [11]. In addition, the authors studied the catalytic properties of the Rh/Ce_0.75_Zr_0.25_O_2_/Al_2_O_3_/FeCrAl composite catalyst with respect to the conversion of diesel fuel into synthesis gas and concluded that it can be used in fuel cell power generators [10].

Using advanced materials and methods for environmentally protective purposes was also within the scope of this Special Issue. For instance, waste produced by the textile-industry contains various dyes (e.g., methyl orange, methylene blue, and rhodamine B) that pose environmental risks. In the research presented in [18], the authors synthesized zerovalent iron (ZVI) using biological reducing agents isolated from tea-leaves (polyphenolic compounds) and showed the applicability of the designed ZVI system to wastewater treatment. In [16], the photodegradation of methylene blue over BNO_x_ catalysts (containing 4.2 and 6.5% oxygen) was studied in detail. The authors revealed the important role of surface oxygen defects in the adsorption capacity and photocatalytic activity of BNO_x_.

The Editors of Special Issue are very grateful to all the authors for their excellent contributions and to the editorial team of *Materials* for their kind assistance and peer review. The readers will definitely enjoy this Special Issue and, hopefully, find new ideas and concepts for their future investigations!
